# Elemental Contrast X-ray Tomography Using Ross Filter Pairs with a Polychromatic Laboratory Source

**DOI:** 10.1038/s41598-017-00304-7

**Published:** 2017-03-16

**Authors:** Benedicta D. Arhatari, Timur E. Gureyev, Brian Abbey

**Affiliations:** 1 ARC Centre of Excellence in Advanced Molecular Imaging, Department of Chemistry and Physics, La Trobe University, Victoria, 3086 Australia; 2 ARC Centre of Excellence in Advanced Molecular Imaging, School of Physics, The University of Melbourne, Parkville, VIC 3010 Australia

## Abstract

The majority of current laboratory based X-ray sources are polychromatic and are not tuneable. This lack of monochromaticity limits the range of applications for these sources and in particular it reduces the elemental specificity of laboratory based X-ray imaging experiments. Here we present a solution to this problem based on the use of Ross filter pairs. Although such Ross filter arrangements have been applied in proof-of-principle spectroscopy experiments, to date there have been no reports of this approach used for full-field X-ray imaging. Here we report on the experimental demonstration of Ross filter pairs being used for quasi-monochromatic, full-field imaging. This arrangement has several important benefits for laboratory based X-ray imaging including, as we demonstrate, elemental contrast enhancement. The method is demonstrated both for two-dimensional radiography and for three-dimensional X-ray tomography.

## Introduction

Polychromatic laboratory based X-ray sources are used the world over for X-ray imaging experiments on a wide range of life and materials science samples^1, 2^. Although monochromatic, high-brightness and energy-tuneable sources of X-rays, such as synchrotrons do exist, access to them is still relatively limited. This has driven research into a range of alternative, more compact laboratory versions capable of mimicking some of the key characteristics of synchrotrons and yet currently the vast majority of laboratory sources still have a very broad spectral bandwidth. One of the key applications that are bandwidth limited is elemental contrast imaging. Elemental contrast imaging is routinely used to map the spatial distribution of individual elements in compound samples and is the subject of widespread interest in many frontier research fields^3–5^. A number of alternative spectroscopic methods for elemental or biochemical mapping are available such as Fourier Transform Infrared (FTIR) spectroscopy or the Time of Flight Secondary Ion Mass Spectroscopy (ToF-SIMS). However, these types of optical spectroscopy based techniques are only applicable for the identification of elements located at or near the sample surface. In the case of thick, optically opaque compound samples elemental distribution maps can be obtained primarily in two different ways: through the analysis of the characteristic peaks present in X-ray fluorescent (XRF) spectra^6, 7^ and by analysing the sample absorption properties at different energies^8, 9^, which is known as K-edge subtraction (KES). Firstly, X-ray fluorescence microscopy is well-established as a powerful and precise tool for obtaining elemental distributions in both two^10^ and three dimensions^6^. However, spatially-resolved XRF maps are normally generated via scanning a small focused X-ray spot on the sample which can be time-consuming compared to full-field approaches. In addition, XRF still has some limitations with respect to sample thickness due to the weak fluorescent signal and self-absorption effects which can lead to distortion of the measured spectra^11^. These artefacts are particularly apparent in the case of low-Z elements, where the low energy fluorescent X-rays can be severely attenuated by the sample to the point where it may no longer be measurable. Secondly, K-edge subtraction (KES) is a popular full-field imaging technique for obtaining the distribution of a specific element in a compound sample by subtraction of two images collected either side of an absorption edge. Mapping of the elemental distribution has also been developed based on phase-contrast imaging^12^ involving taking phase measurements in the vicinity of the elemental absorption edge of interest. Alternatively, for quantitative purposes, elemental or compositional mapping of three-dimensional data can be performed using sophisticated software by segmentation process^2, 13, 14^. More importantly, most of the work on full-field elemental mapping has been carried out using synchrotron sources^6–10^. Unfortunately, there are significant barriers, to carrying over these methods to the laboratory, including a polychromatic illumination, lower incident flux and correspondingly longer exposure times. However, the ease of access to laboratory sources compared to synchrotrons still makes them highly attractive for elemental contrast imaging experiments. Therefore, methods which allow researchers to utilise polychromatic x-ray sources for elemental contrast imaging have the potential to be of enormous benefit to a large number of laboratories worldwide.

Two other approaches which are utilised to enhance elemental contrast in laboratory-based imaging are dual energy CT (Computed Tomography)^15^ and X-ray tomography using energy-resolved area detectors^16, 17^. To place the current approach using Ross filter pairs in context we now compare and contrast these two techniques using the current method. Dual energy CT is a well-established method for differentiating between sample components, including bone and soft tissue, based on their specific attenuation^18–20^. This can be done by using different tube voltages or by using two layers of detectors with different energy responses^21^. An advantage of the dual-energy scanning approach compared with the current method are that incident energy can often be varied, for example by changing the tube voltage. However, the data analysis for dual-energy is often quite complex, involving either a pre- or post-processing step. To properly interpret the data an extra calibration step is normally required tailored to the specific materials under investigation. The extra calibration step also depends on the characteristics of the incident spectrum and response of the detector over a relatively broad range. A second emerging technique which shows great promise for elemental enhancement is the use of energy-discriminating X-ray area detectors, which are becoming increasingly prolific as tools for spectroscopic imaging using laboratory-based sources for material differentiation^22^. In this approach multiple energy thresholds are used to separate out particular elements^23, 24^. Because these detectors fundamentally rely on direct photon detection to accurately discriminate the measured signal in terms of energy, the effective resolution of these detectors is limited by the actual pixel size on the chip. Currently the smallest pixels available in commercial energy-resolving area detectors have a pitch of 60 µm^25^. In both the dual-energy CT and energy-resolving X-ray detector approach the resolution of the thresholds defining the windows can be rather limited. In the former the case of dual-energy CT (e.g. by changing the tube voltage), it is as a result of relying on the difference between two broad spectra. This makes defining discrete energy windows for complete separation of elements challenging. In the case of energy-resolving X-ray area detectors the energy threshold resolution is typically 1–2 keV due to the effects of charge sharing^25^. Fortunately, a hyperspectral X-ray detector with an energy resolution of less than 1 keV has been recently introduced^26^. This new detector has the capability for studying the distribution of catalyst material and mapping mineralised ore within the sample. However, currently this detector has rather large pixel pitch of 250 µm; so the improved energy-resolution comes at the cost of spatial resolution. Generally, this is a feature of spatial and energy-resolving X-ray detectors; i.e. that the available pixel size for detectors that can energy-threshold is larger than can be achieved with conventional scintillator coupled X-ray detectors. Depending on the available geometry, this places constraints on the resolution that can be achieved in an X-ray CT experiment.

In summary, the advantages of using Ross filter pairs for elemental separation in X-ray CT compared to the other methods described are that the filters absorption edges provide a very well-defined and sharp energy window. In addition, from a data analysis point of view the technique is very simple, requiring no complex pre or post processing of data. Finally, the spatial resolution is identical to regular X-ray CT with no impact caused by the presence of the filters. The disadvantage of Ross filters is that one needs to carefully select the pairs depending on the elements that need to be separated. In addition, in order to separate multiple elements, multiple different filter pairs need to be used. Some of these limitations could be addressed in future via use of a ‘filter wheel’ containing multiple filters. However, each of these techniques have their complementary strengths and weaknesses depending on the specific application.

Elemental contrast imaging using Ross-pair filters, without the need for a sophisticated and expensive detectors or complex pre/post processing provides the primary motivation for the present study. The use of Ross-pair filters for producing monochromatic, tuneable X-rays from a polychromatic source has previously been demonstrated in the context of X-ray spectroscopy^27, 28^. Here, we extend this idea to full-field X-ray imaging, demonstrating that the concept enables elemental mapping in both two and three dimensions. The basic principle relies on choosing filters with absorption edges located above and below the absorption edge corresponding to the element of interest^29^. In this article the method for using Ross filters for full-field elemental X-ray contrast imaging with a polychromatic laboratory source is presented. Our results show that with the correct choice of filters excellent contrast can be obtained for the element of interest paving the way to Ross filters being routinely used in full-field laboratory based X-ray imaging with elemental sensitivity. Therefore, this technique will likely have a number application in a wide range of scientific research areas, especially in material science applications and coating industry. Other applications where the elements may be known, but the distributions hard to obtain include rare earth samples and battery materials.

## Results

**Figure 1:**
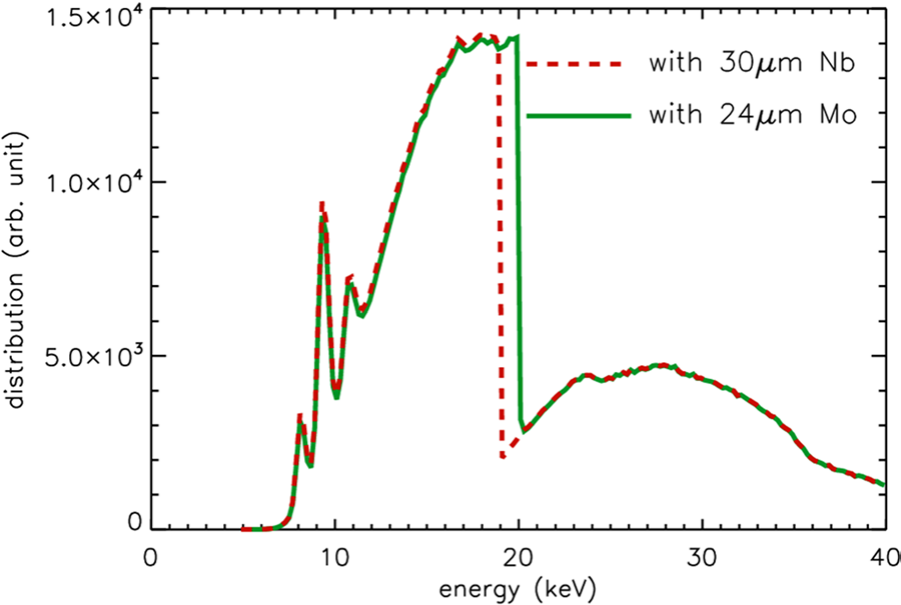
Ross filter pair transmission spectrum. Simulation of X-ray transmission through a Ross filter pair, *∆E*_−_, comprised of Nb and Mo foils is presented as a function of the X-ray energy. The naked beam spectra, *S*(*E*), was measured experimentally. The green solid line shows the transmission spectra from Mo filter, while the red dashed line from Nb filter. Both lines are matched perfectly outside of the K-edge energy interval.

Two narrow X-ray spectral bandwidths were defined through the combination of a set of Ross filters pairs, with each pair comprising two elements of slightly different atomic number. The bandwidth of the pairs was determined by the K-edge energy difference between the two elements that made up the filter. The thickness of each pair (Table 1) was chosen such that the X-ray transmission was essentially equal for all photon energies above and below the designated photon energy band. This is illustrated in Fig. 1. By matching the X-ray transmission for the two different elements within the Ross filter pair either side of the relevant absorption edges, the resultant subtracted image contains mainly the contributions from X-ray energies within the defined bandwidth with very little residual signal from the other energies.

**Table 1:** Key parameters for Ross filter pairs used in this study.

Ross Filter Pair	Elements	Thickness (μm)	Atomic Number	K-edge Energy	Spectral Bandwidth
Δ*E*_*−*_	Nb	30 μm ± 15%	41	18.99 keV	1.01 keV
	Mo	24 μm ± 15%	42	20.00 keV	
Δ*E*_+_	Pd	25 μm ± 15%	46	24.35 keV	1.17 keV
	Ag	27 μm ± 15%	47	25.52 keV	

Thickness tolerance is provided by the manufacturer (Goodfellow Inc.).

The upper and lower bands are determined by the K-edge energy of the filter pairs. Figure 1 shows a 1 keV spectral bandpass created by a Ross filter pair containing a 30 μm thick niobium (Nb) and a 24 μm thick molybdenum (Mo) foil. The lower band is at 18.99 keV and upper band is at 20.00 keV. The number of photons in the energy bandwidth is given by the following integral:

1 $${N}_{{\rm{\Delta }}{E}_{-}}={\int }_{{K}_{Nb}}^{{K}_{Mo}}S(E)dE$$

where *N*_*∆E*__ is the number of photons within the spectral bandwidth of the Ross filter pair, *K*_*Nb*_ and *K*_*Mo*_ are the K-edge energy of Nb and Mo respectively and *S*(*E*) is the characteristic X-ray spectrum of the source expressed in the number of photons per energy interval. In Fig. 1, *S*(*E*) was characterised using an energy-dispersive detector (for more information on the spectrum measurements, please see the methods section). For the simulations, the Nb and Mo filters, each with a specific thickness, were applied to the measured spectral data based on their known linear attenuation coefficients^30^.

The ∆*E*_−_ spectral band is tailored such that a narrow distribution of energies is produced just below the absorption edge of the element of interest. When using a monochromatic tuneable X-ray source, typically only a single monochromatic (e.g. ∆*E/E* < 10^−4^) image is required below the absorption edge. Conversely, with a polychromatic source, a narrow bandwidth of energies is used to approximate a single monochromatic energy. This spread of energies necessitates that two separate images with slightly different cut-off energies, both lying below the absorption edge of the element of interest, are subtracted. The resultant image produced from the subtraction has an effective bandwidth which is then narrow enough to be used for elemental studies. Our method could be then considered an analogue of KES, using the Ross filters as a means of obtaining the monochromatic images. A second Ross filter pair, with a spectral band, ∆*E*_+_, is required to represent the single monochromatic energy lying above the absorption edge of interest. In the present study this second Ross filter pair comprised of 25 μm palladium (Pd) and 27 μm silver (Ag). In summary then, to reproduce the tuneable monochromatic experiment for elemental contrast enhancement using a polychromatic source and two Ross filter pairs one requires four filters and four separate images (as opposed to two for monochromatic X-rays). A summary of the filters used and their corresponding thicknesses are given in Table 1.

**Figure 2:**
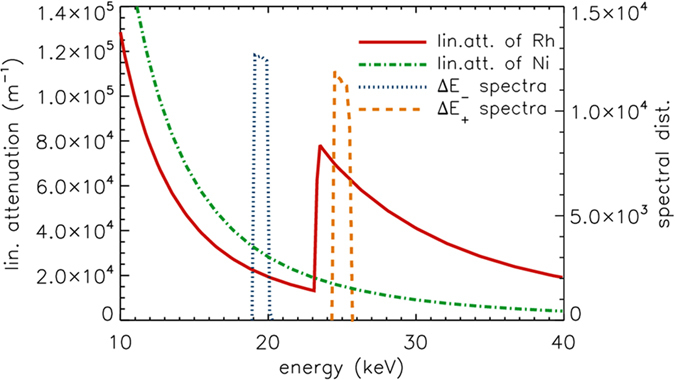
X-ray attenuation behaviour of Rh and Ni. Linear attenuation coefficient of rhodium and nickel as a function of energy are plotted together with the Δ*E*_−_ and Δ*E*_+_ of the Ross filter pairs. The K-edge energy of Rh (red solid line) sits between low-, Δ*E*_−_, and high spectral band, Δ*E*_+_, while it is not the case for Ni (green dashed dotted line).

To demonstrate the application of Ross filter pairs to elementally enhanced full-field X-ray imaging, a compound sample was imaged. This sample was composed of 12 μm thick rhodium (Rh) and 18 μm thick nickel (Ni) foils. Rhodium has a K-edge energy of 23.2 keV, which lies in between the maximum energy within Δ*E*_*−*_ (defined by the Mo edge) and the minimum energy within Δ*E*_+_ (defined by the Pd edge). Nickel meanwhile has a K-edge at 8.3 keV, well away from the absorption edges of the elements which make up the two Ross filter pairs. The X-ray transmission behavior of Rh and Ni is shown overlaid by the two energy bandwidths Δ*E*_*−*_ and Δ*E*_+_ in Fig. 2. As can be seen from this figure, the use of two Ross filter pairs above and below the absorption edge of Rh is designed to mimic the effect of using two monochromatic energies in a conventional absorption edge enhanced imaging experiment or KES.

**Figure 3:**
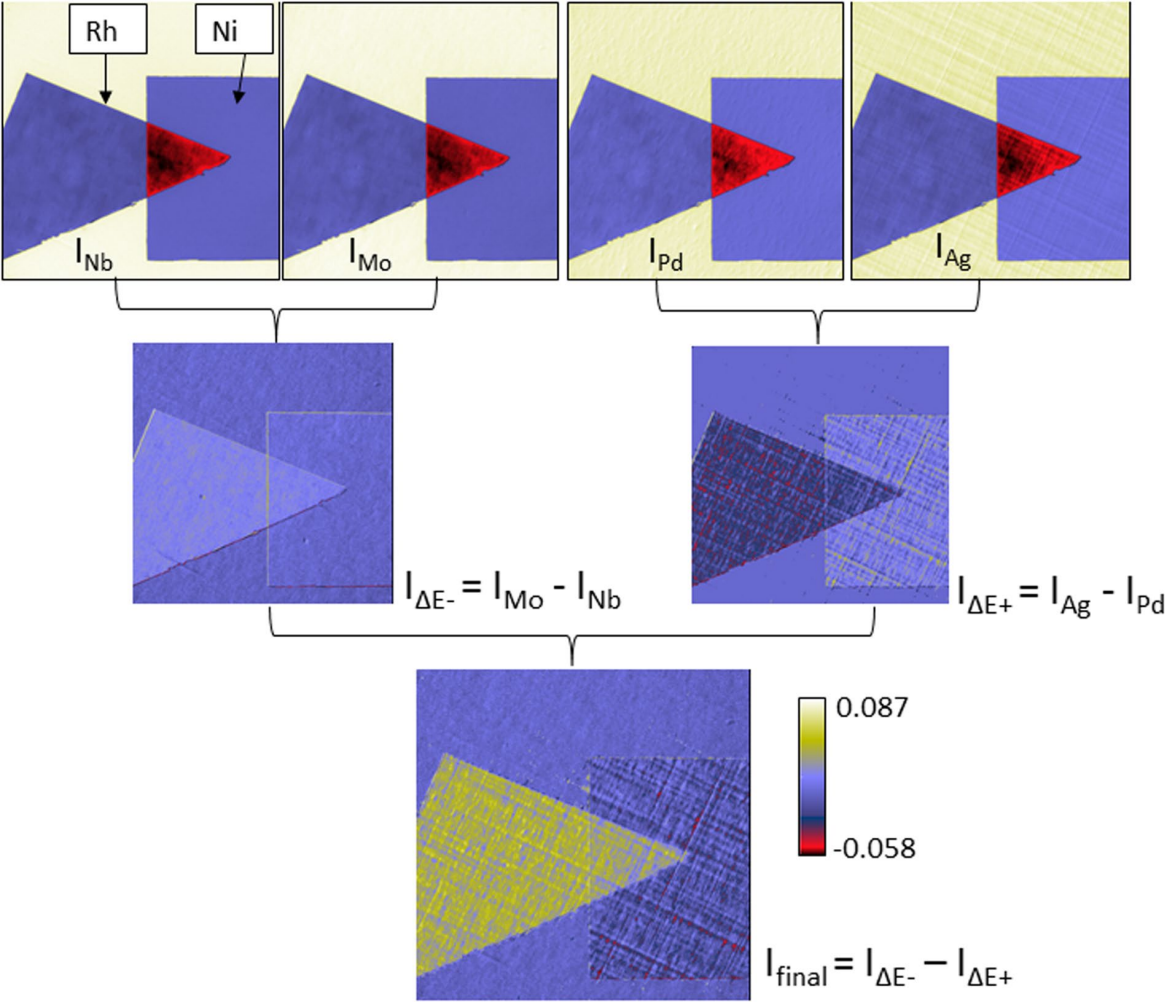
Elemental contrast imaging flowchart. Flowchart illustrates the process of obtaining the elemental distribution of Rh. Top part shows four intensity images taken with Nb, Mo, Pd and Ag filters respectively. The resultant subtracted intensities corresponding to low and high energy band are showed in the second row. Bottom row shows the final result of the process.

X-ray transmission data were recorded with the Ross filter pairs placed immediately after the X-ray source. A complete set of two-dimensional elementally-enhanced data consisted of four separate intensity images, one for each of the four elements comprising the two filter pairs. Each image was corrected for any non-uniformities introduced by the imaging system and by the filter, by taking a reference image of the illumination plus filter but with the sample removed. Each image contained 512 × 512 pixels with an effective pixel size of 11 μm. The intensity images are shown in the top part of Fig. 3. A qualitative assessment of the four images reveals no obvious differences in contrast between the two objects in the data, with both Rh and Ni objects having similar grey levels in the images. From this we conclude that in a conventional polychromatic X-ray imaging experiment it would be impossible to distinguish the two objects on the basis of their elemental composition. In the raw images using Pd and Ag filters, with the sample in place there are a small amount of stray scattered photons which contributed a background signal across the image. To remove this effect, we set a pedestal threshold equal to 2% of the maximum intensity value on both images. The resultant intensities corresponding to Δ*E*_*−*_ and Δ*E*_+_ after image subtraction, which are labelled ${I}_{{\rm{\Delta }}{E}_{-}}$ and ${I}_{{\rm{\Delta }}{E}_{+}}$ respectively, are shown in the second row of Fig. 3. Note that the subtraction is carried out on a logarithmic scale in order to obtain the linear absorption properties. Unlike the individual intensity images, the images produced by the two Ross filter pairs produce a striking contrast between the Rh and Ni objects. Specifically the transmitted signal for the element of interest (Rh) changes significantly between ${I}_{{\rm{\Delta }}{E}_{-}}$ and ${I}_{{\rm{\Delta }}{E}_{+}}$, while the Ni object has a similar transmission within the two bandwidths. Subtraction of ${I}_{{\rm{\Delta }}{E}_{+}}$ from ${I}_{{\rm{\Delta }}{E}_{-}}$ then leaves a single image which is dominated by the signal from Rh, though there is clearly a residual absorption- and scattering signal from the Ni, it is a relatively small perturbation on the Rh object. This effect is commonplace in monochromatic absorption edge enhanced contrast imaging, but to the authors knowledge this is the first time that such contrast enhancement had been achieved using Ross filter pairs and a polychromatic X-ray source. Our experimental results are entirely consistent with the data presented in Fig. 2 showing that the variation in the absorption coefficient for Ni is approximately linear, with no discontinuities present between the two energy bandwidths defined by the Ross filters. The final image $({I}_{final}={I}_{{\rm{\Delta }}{E}_{-}}-{I}_{{\rm{\Delta }}{E}_{+}})$ gives a high-quality map of the elemental distribution of Rh.

**Figure 4:**
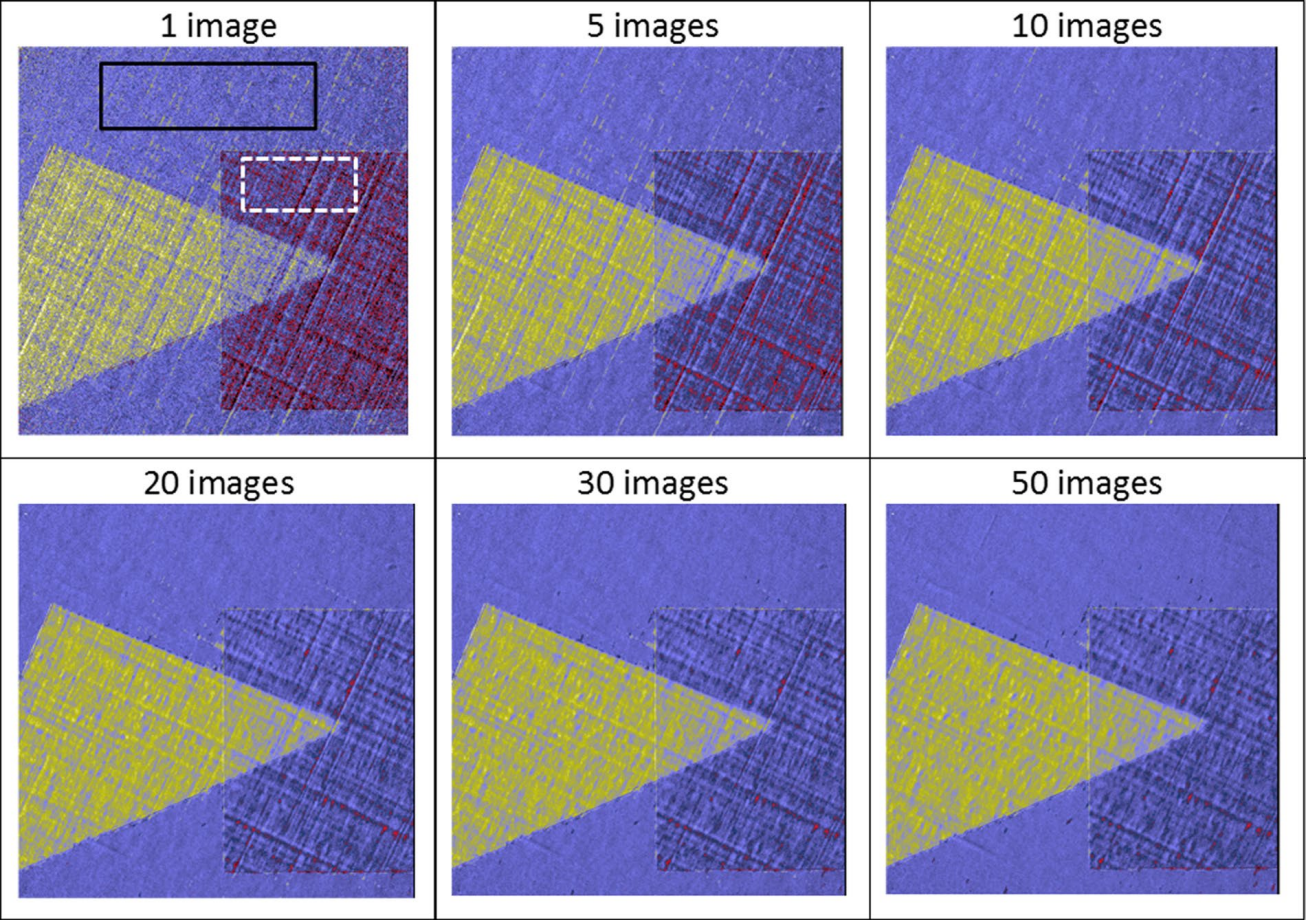
Elemental contrast vs the number of images averaged. Variation of the final image, *I*_*final*_, are shown as a function of the number of images averaged. Solid rectangle area represents the location of the background to calculate SNR, while white dashed rectangle for error in residue calculation, *ε*_*res*_.

In order to investigate the effect of statistics on the resulting signal-to-noise ratio (SNR) of the elemental distribution map, different numbers of intensity images were averaged. Figure 4 summarises the results of averaging 1, 5, 10, 20, 30 and 50 separate images. The linear artefacts are present when the films used as filters are not smooth. In the present case there were linear striations in the Ag thin film we used as a filter because of the way it was manufactured. The use of a less highly textured filter would avoid this problem altogether in the future. Alternatively, rotating the filter during the data acquisition may possibly reduce such artefacts. The SNR level for each result was calculated^31^ using the following formula:

2 $${\rm{SNR}}=\frac{|\langle {I}_{Rh}\rangle -\langle {I}_{background}\rangle |}{\sqrt{{\sigma }_{Rh}^{2}+{\sigma }_{background}^{2}}}$$

where $\langle \rangle$ indicates the average value whilst *σ* is the standard deviation. The black rectangle defined by the solid line in the left pane of Fig. 4, encloses the area used to represent the background data. The white dashed line defines an area where we anticipate that residue from the Ni foil may persist in the Rh elemental map. The percentage approximation error of the residue is calculated^8^ as follows:

3 $${\varepsilon }_{res}=\frac{|\langle {I}_{residue}\rangle |}{\langle {I}_{Rh}\rangle }\times 100 \% $$

**Figure 5:**
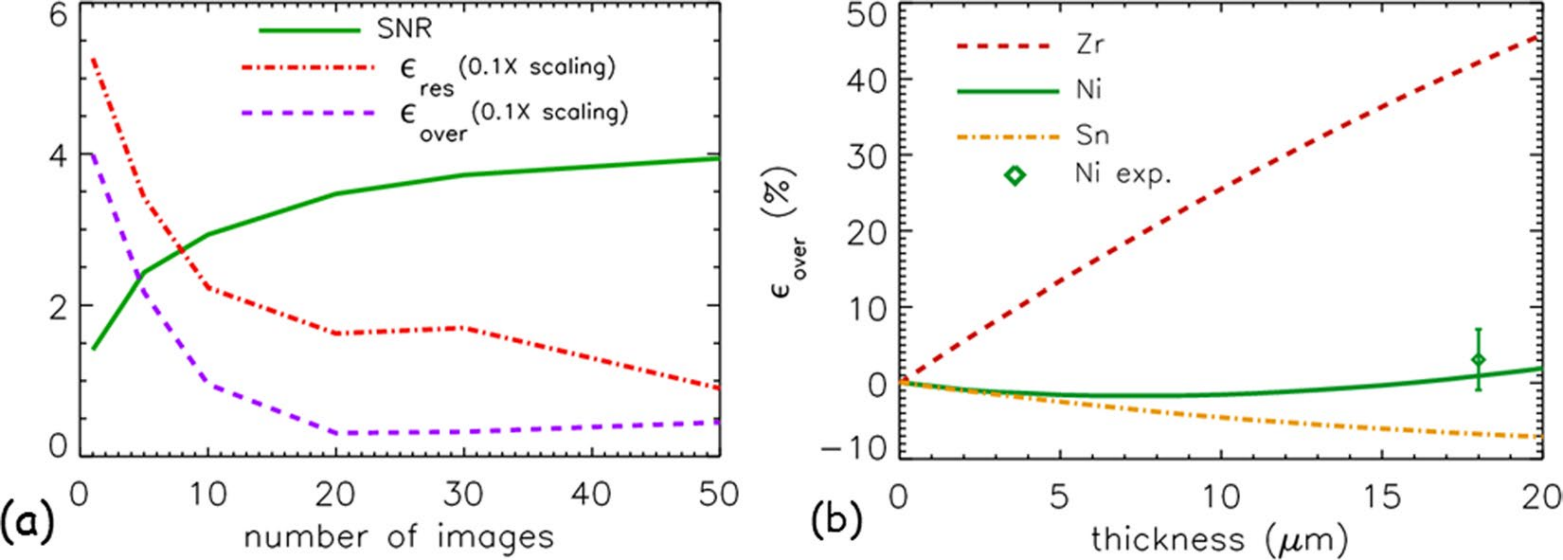
SNR, *ε*_*res*_, and *ε*_*over*_ evaluation. Plot summarising the calculated SNR, *ε*_*res*_ and *ε*_*over*_ is presented in (**a**) as a function of number of images averaged. (**b**) as a plot of the Rh percentage change, *ε*_*over*_, in the overlapping region as a function of thickness of other material.

The values of the SNR and ${\varepsilon }_{res}$ respectively are plotted as a function of number of images averaged in Fig. 5(a). The higher the number of images used in the averaging step, the higher is the SNR in the final image. On the other hand, the error percentage of the residue becomes smaller when higher number of images are averaged. The variation of the elemental distribution map in the overlapping region between Rh and Ni is also evaluated as follows:

4 $${\varepsilon }_{over}=\frac{\langle {I}_{Rh}\rangle -\langle {I}_{Rh+Ni}\rangle }{\langle {I}_{Rh}\rangle }\times 100 \% $$

The percentage change, *ε*_*over*_, is shown by the purple dashed line in Fig. 5(a). The result shows that the elemental distribution of Rh in the overlapping region is recovered more effectively when 20 or more images are averaged. The variation of the elemental distribution of Rh in the overlapping region in comparison to other elements (Zr, Ni and Sn), is also evaluated in the simulation study as a function of their thickness. The result is shown in Fig. 5(b). The experimental value of *ε*_*over*_ for the Ni foil, obtained from the average of 20 images, is included in the plot of the simulated data in Fig. 5(b). The Ni foil experimental data point (*ε*_*over*_ = 3 ± 4%) is consistent with the predicted value of *ε*_*over*_ = 1%. The plot shows that *ε*_*over*_ is strongly dependent on the absorption properties of the other overlapping element. Elements within the sample (in this case Zr) which have a K-edge energy position close the absorption edges of the elements which make up the two Ross filter pairs will absorb X-rays more strongly. By contrast elements whose edges are positioned far away the K-edges of the filters absorb less strongly. Hence, whilst distinguishing between two elements, both having K-edges within the energy bandwidth defined by the filter pairs may be difficult, this effect will increase the relative contrast of elements whose K-edges are close together above what is achieved using the whole polychromatic source spectrum.

**Figure 6:**
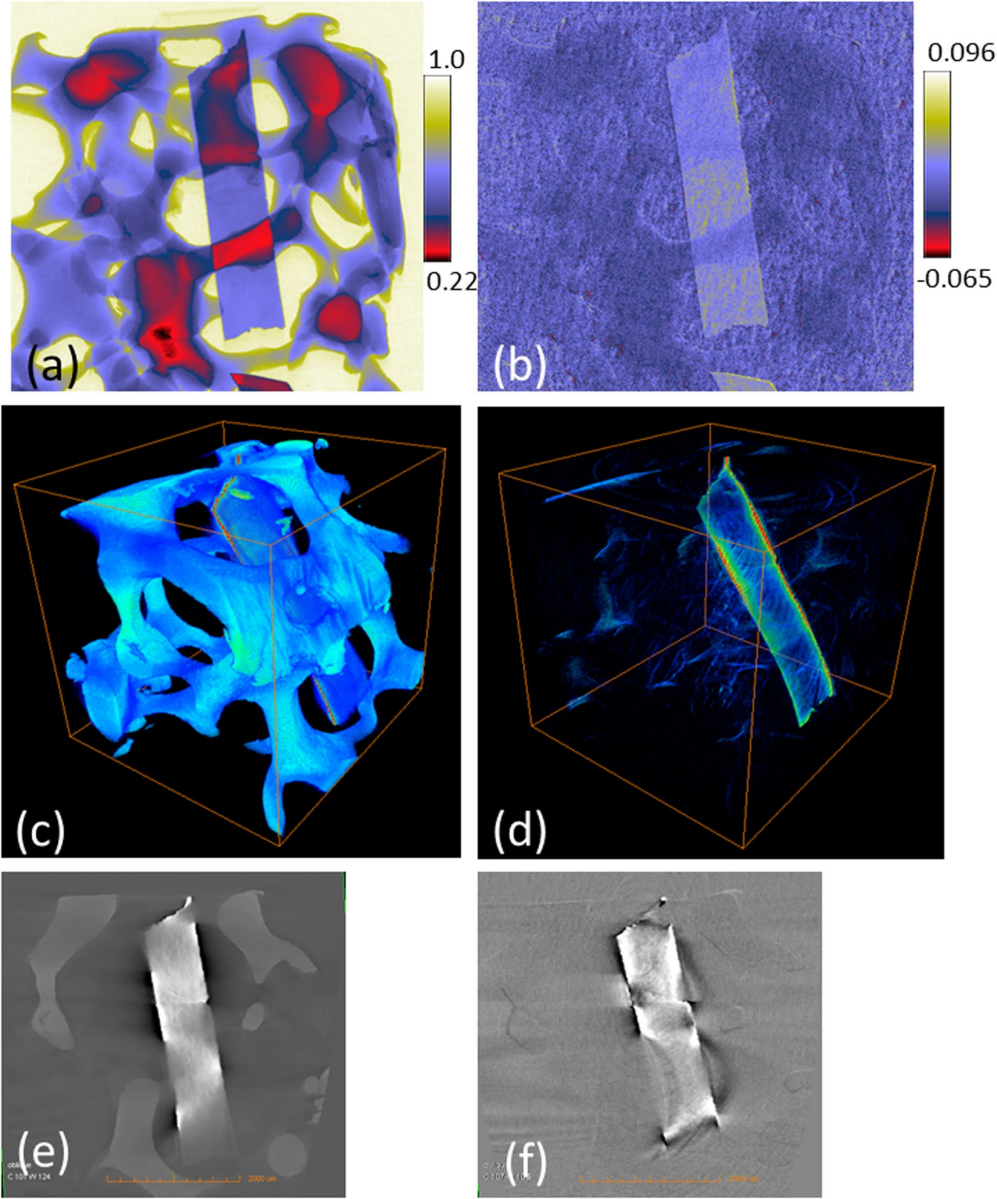
Three-dimensional elemental contrast demonstration. (**a**) and (**b**) shows one projection image before and after elemental contrast enhancement. Three-dimensional reconstructed image without applying elemental contrast is shown in (**c**) with the corresponding CT slice in (**e**). The elemental contrast method applied to each projection is shown in (**d**) as a three-dimensional reconstructed image of Rh material with the corresponding slice in (**f**).

Elemental contrast imaging using the Ross filter arrangement can be readily extended to three-dimensional (3D) imaging. The tomographic sample in this case consisted of a piece of Rh inserted into aluminium foam cube forming a test 3D object. The method described above for the elemental contrast enhancement based on four intensity measurements, was then applied to images collected at each of the 181 projection angles. This resulted in four sets of tomographic data collected using Mo, Nb, Pd and Ag filters respectively. One of the projections taken using Mo filter is shown in Fig. 6(a). Figure 6(b) shows the same projection image after the elemental contrast enhancement process. The difference between the last two results is striking; in Fig. 6(b) the Rh component is obvious, whilst it is not distinguishable at all from the Al in Fig. 6(a) where the elemental contrast method was not applied. The corresponding three-dimensional reconstruction result is also displayed. The standard polychromatic X-ray tomography result with Mo filter in place is shown in Fig. 6(c) whilst the elementally enhanced tomography reconstruction is displayed in Fig. 6(d). The corresponding slice images are shown in Fig. 6(e) and (f) respectively. As with the two-dimensional result, there is still a small residual signal from the Al even after subtraction. However, this residue is a small when compared to the contrast of the element of interest. The presence of metal streak artefacts, which could not be removed, and which cause a dark streak region around the high density material are apparent in Fig. 6(f). In addition, a beam hardening artefact is also present that causes a visible bright residue around the Rh. However, these types of artefacts are not the result of the Ross filter method, and post-processing methods have been developed to try to correct for these^32, 33^.

## Discussion

We have shown that the Ross filter arrangement provides an excellent tool for achieving elemental contrast imaging using a laboratory-based polychromatic X-ray source. This technique can achieve micron-size spatial resolution which, due to the large pixel size, is currently challenging with energy-resolved area detectors. In addition, compared to dual-energy CT, the filter pairs provide two sharp-edged energy windows enabling a relatively narrow bandwidth (<0.5 keV) which is not generally possible using dual-energy CT. Since dual-energy CT normally generates only two images (at ‘high’ and ‘low’ energy) it cannot be used to create separate images of the sample above and below specific absorption edges. Therefore, on the basis of simple subtraction of two energy spectra, the Ross-filter pairs should outperform dual-energy CT in terms of separating out certain elements. However, using more complex polynomial functions in combination with dual-energy CT and applying the appropriate weighting factor for individual pixels in an iterative fit procedure, has been shown to yield far better results than simple subtraction. A detailed experimental comparison of the effectiveness of this two different approaches will be pursued in future work.

In addition, the Ross filter pair arrangement demonstrated here shows promise as a novel, but simple approach to achieving elemental contrast in 2D- and 3D images. However, as with any approach to elemental X-ray imaging there are also limitations on using this approach. At present we have only tested the technique for inspecting one element with the K-edge energy inside the two Ross-pair filters. If the other element has also a K-edge inside energy bandwidths of the two Ross-pair filters such as Rh and Ru (Ruthenium), then the level of discrimination between the two elements will dramatically decrease (perhaps even to a similar contrast level as can be achieved without the filters in place). In addition, the Ross filter pair arrangement cannot be used to inspect materials with the same element as the element of the Ross filter itself. Therefore, some prior knowledge of sample compositions is required. Moreover, some elements in the periodic table are not available in the form of foils which places constraints on which Ross filter pairs can be created. Nonetheless there are many applications, where the elemental composition is known and where a very simple analysis approach giving both good elemental contrast and high-spatial resolution is desirable. For these types of applications (e.g. batteries, mineral deposits, etc.) Ross-filter pairs could be the ideal solution.

In summary, this Ross-filter technique appears both novel and promising. In the first experiment, four different filters with specific thicknesses were used to create two Ross filter pairs. Each Ross filter pair creates a narrow bandwidth that approximates a monochromatic image below or above the absorption edge of the material of interest, respectively. The method was extended to tomography demonstrating that the same benefits can be achieved in three-dimensional elemental imaging. Further work needs to be carried out to quantify, for example the detection limit for low elemental concentrations. Having now established the fundamental principles, the next step will be to apply this technique to realistic samples such as catalyst materials, battery materials, coating, etc. Nevertheless, from this first demonstration of full-field elemental contrast X-ray tomography using Ross filters this new approach appears very promising.

## Methods

### X-ray radiography and tomography

A laboratory-based polychromatic X-ray source, Xradia© micro XCT200 (Carl Zeiss X-ray Microscopy, Inc.) was used for X-ray imaging in this study. XCT200 uses a microfocus X-ray source with a rotating sample holder and a high resolution imaging detector system. The source consists of a closed X-ray tube containing tungsten as a target material with a tube voltage of 40 kV and a peak power of 10 W. The imaging detector comprised an X-ray scintillator optically coupled to a CCD camera via a 4× objective lens. Each image comprises 512 × 512 pixels with an effective pixel size of 11 μm. The exposure time for each image was 15 seconds. For the tomographic measurements, the sample was rotated whilst recording transmission X-ray data. One complete tomographic data set using a single filter comprised 181 equiangular projections taken in 1 degree steps. A filtered back-projection algorithm (using the TXMReconstructor software, Carl Zeiss X-ray Microscopy, Inc. USA) was used to reconstruct a three-dimensional image from the two-dimensional projection data set. The TXM3DViewer (Carl Zeiss X-ray Microscopy, Inc. USA) was used for 3D visualization.

### X-rays spectral measurement

The characteristic X-ray spectrum of the source, *S* (*E*), was measured using an energy dispersive detector (XR-100T-CdTe, AMPTEK Inc.). In the measured energy range, the detection efficiency of CdTe is approximately 100%^34^. Therefore, the spectral response of the detector is approximately 1 in this energy range. The air absorption in the short path and the thin beryllium entrance window to the detector give negligible effect and can be neglected in the detected histogram providing a good approximation to the source spectrum. The histogram contains narrow peaks corresponding to the characteristic x-ray energies of the Tungsten target and a wide continuous energy spectrum (Bremsstrahlung) with a cut-off energy of 40 keV. The measured spectrum has a low energy cut-off as a result of the detector settings.
